# Intake or Blood Levels of Magnesium and Risk of Metabolic Syndrome: A Meta-Analysis of Observational Studies

**DOI:** 10.3390/nu17101667

**Published:** 2025-05-14

**Authors:** Youngyo Kim, Youjin Je

**Affiliations:** 1 Department of Food and Nutrition/Institute of Agriculture and Life Science, Gyeongsang National University, Jinju 52828, Republic of Korea; youngyokim@gnu.ac.kr; 2 Department of Food and Nutrition, Kyung Hee University, Seoul 02447, Republic of Korea

**Keywords:** dietary magnesium, serum magnesium, magnesium status, metabolic syndrome, meta-analysis, systematic review

## Abstract

Background/Objectives: The association between magnesium and metabolic syndrome has not been comprehensively examined. We conducted a meta-analysis to quantitatively evaluate the association between intake and blood levels of magnesium and metabolic syndrome. Methods: We searched PubMed, Scopus, and ISI Web of Science databases to identify studies reporting an association between magnesium and metabolic syndrome up to April 2025. To pool the effect sizes on metabolic syndrome according to intake and blood levels of magnesium, a random effects model was used. Results: Twenty-seven publications including 95,933 participants were included in the meta-analysis. The relative risk summary of metabolic syndrome for highest versus lowest intake of magnesium was 0.79 (95% confidence interval [CI]: 0.71–0.88) for prospective cohort studies. In the meta-analysis of cross-sectional studies, magnesium intake was inversely associated with metabolic syndrome (odds ratio = 0.61; 95% CI: 0.39–0.94). High blood levels of magnesium were inversely associated with metabolic syndrome (effect estimate = 0.53; 95% CI: 0.37–0.76). Conclusions: The present meta-analysis indicated that magnesium intake was inversely associated with a risk of metabolic syndrome. Regarding the association between blood levels of magnesium and metabolic syndrome, a significant inverse association was found, but the interpretation was cautious due to the observed high heterogeneity. The association between magnesium status and metabolic syndrome needs to be confirmed with further prospective studies.

## 1. Introduction

Magnesium is a macromineral contained in green leafy vegetables, legumes, beans, peas, nuts, and whole grains [1]. As the second most abundant cation and fourth most plentiful mineral in the human body, magnesium is involved in more than 600 metabolic reactions [2,3]. Magnesium maintains deoxyribonucleic acid (DNA) stability by forming a structure of DNA and protecting DNA from oxidative stress [3]. Also, magnesium plays a vital role in protein synthesis and acts as a key regulator of cell cycle progression and cell proliferation [3,4]. It is essential to maintain the homeostasis of magnesium in the bones, intestines, and kidneys because magnesium plays a diverse physiological role in the brain, skeletal muscles, and heart [3]. Magnesium deficiency can increase the risk of a variety of chronic diseases by affecting the normal functioning of body functions that depend on magnesium [2].

Metabolic syndrome is the co-occurrence of at least three abnormal levels for waist circumference, fasting blood glucose, blood triglyceride, blood pressure, and blood high-density lipoprotein (HDL) cholesterol [5]. Metabolic syndrome, the leading cause of which is insulin resistance, is positively associated with the risk of many chronic diseases, including cancer, cardiovascular disease (CVD), non-alcoholic steatohepatitis, neurodegenerative disorders, and chronic kidney disease [6]. Worldwide, metabolic syndrome has a high prevalence rate of over 10%, ranging from 12.5% to 31.4% depending on the definition criteria applied [7]. There are many lifestyle risk factors for metabolic syndrome and dietary factors are considered vital among them [8,9,10].

Regarding minerals, accumulating evidence has shown that a high sodium status is associated with an increased risk of metabolic syndrome [11]. Many previous observational studies have explored the risk of metabolic syndrome in relation to the intake [12,13,14,15,16,17,18,19,20,21,22,23,24,25,26,27] or blood levels [27,28,29,30,31,32,33,34,35,36,37,38] of magnesium. Although there have been previous attempts to integrate evidence on the association between magnesium intake and the risk of metabolic syndrome, most of the included studies were cross-sectional [39,40]. As several cohort studies have recently reported the results of studies on magnesium intake and metabolic syndrome [23,24,25], there is a need to integrate the prospective association between magnesium intake and metabolic syndrome. Therefore, we conducted a systematic review and meta-analysis of observational studies to summarize the evidence of the association between intake and blood levels of magnesium and metabolic syndrome.

## 2. Materials and Methods

### 2.1. Data Sources and Searches

Electronic databases, including PubMed, Scopus, and ISI Web of Science, were searched to identify articles related to the association between intake or blood levels of magnesium and metabolic syndrome up to April 2025. The search terms used in the database search were as follows: “magnesium” OR “Mg” in combination with “metabolic syndrome”. A manual search was also conducted by reviewing the reference lists of relevant articles to find additional studies. The present meta-analysis was registered at PROSPERO (CRD42024557388).

### 2.2. Study Selection

Studies were selected for the meta-analysis when they met the following criteria: (1) studies with observational designs (cohort, case-control, or cross-sectional designs); (2) studies where intake or blood levels of magnesium were the exposure of interest; (3) studies where the outcome of interest was metabolic syndrome; and (4) studies where the relative risk (RR) or odds ratio (OR) with 95% confidence intervals (CIs) were provided. The studies whose populations consisted only of patients were excluded. When two different articles were from the same study [33,41], we selected the study results with a larger population [33].

### 2.3. Data Extraction

Data were collected from the original articles by two independent authors (K.Y. and Y.J.) following the PRISMA (Preferred Reporting Items for Systematic reviews and Meta-Analyses) statement [42]. The extracted data were the study design, family name of the first author, publication year, geographic region or country, sex of participants, age, sample size, adjusted covariates, exposure category, and RRs and their 95% CI for each exposure category.

### 2.4. Quality Assessment

The quality of studies included in the meta-analysis was examined by a single investigator (Y.K.) and checked by the second investigator (Y.J.). For cohort and case-control studies, a maximum score of 9 was assigned based on the Newcastle–Ottawa scale [43]. The scale consisted of the following three domains: selection of population (0–4 points), comparability for controlling confounders (0–2 points), and outcome ascertainment (0–3 points). A modified form of the Newcastle–Ottawa scale was used to assess the cross-sectional studies, referring to previous studies [44]. Studies with a total score ≥ 8 were rated as high quality.

### 2.5. Statistical Analysis

We used the random effects models of DerSimonian and Laird, which considered both between- and within-study variations [45] to combine RRs from each original study. The RR or OR and its 95% CI were recalculated if an original study did not provide the lowest exposure category as a reference. Statistical heterogeneity and inconsistency among the included studies were assessed using Cochran’s Q test [46] and *I*^2^ statistics [47]. A sensitivity analysis excluding one study at a time was performed to examine the extent to which inferences might be affected by a particular study. Subgroup analyses by sex, geographic region, and sample size were conducted when possible. Begg’s [48] and Egger’s tests [49] were used to evaluate publication bias. All analyses were performed using Stata version 17.0 (STATA Corp., College Station, TX, USA). A two-tailed *p*-value less than 0.05 was considered to be statistically significant.

## 3. Results

### 3.1. Study Characteristics

**Figure 1:**
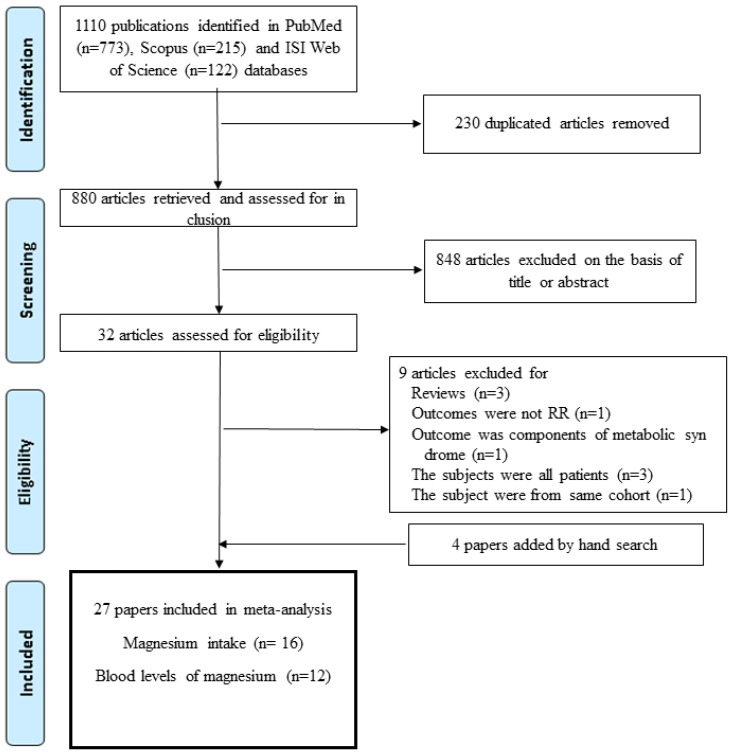
Flowchart for study selection procedure.

Sixteen papers including 74,106 participants and 20,044 cases were eligible for the association between magnesium intake and metabolic syndrome, and twelve papers involving 21,827 subjects were suitable for the association between blood levels of magnesium and metabolic syndrome [12,13,14,15,16,17,18,19,20,21,22,23,24,25,26,27,28,29,30,31,32,33,34,35,36,37,38]. The process for the selection of studies for the meta-analysis is presented in Figure 1. Among the studies included in this meta-analysis, four had a cohort design [14,23,24,25], four had a case-control design [28,29,30,35], eighteen had a cross-sectional design [12,13,15,16,17,18,19,20,21,22,26,27,32,33,34,36,37,38], and one study reported both cohort and cross-sectional results [31]. By region of study, seven were performed in the United States [12,14,15,16,17,20,23], eleven in Asia [21,22,24,25,30,32,34,36,37,38], two in Europe [13,31], five in the Middle East [18,26,27,33,36], and two in Mexico [28,29]. The age range of the participants was 18 or older and the number of subjects in each study ranged from 150 to 9887. All studies were controlled for age, and most studies were adjusted for smoking (n = 17), alcohol (n = 15), and energy intake (n = 14). Most studies defined metabolic syndrome based on the National Cholesterol Education Program Adult Treatment Panel III (NCEP-ATP III) [50], International Diabetes Foundation (IDF) [51], and harmonized [5] definitions. The characteristics of the studies included in the meta-analysis of the association between metabolic syndrome and magnesium intake or blood levels are presented in Table 1, Table 2 and Table 3. As a result of the quality assessment, 24 out of 27 studies scored 8 points or higher; the remaining 3 scored 7 points, showing relatively high quality.

### 3.2. Magnesium and Metabolic Syndrome

**Figure 2:**
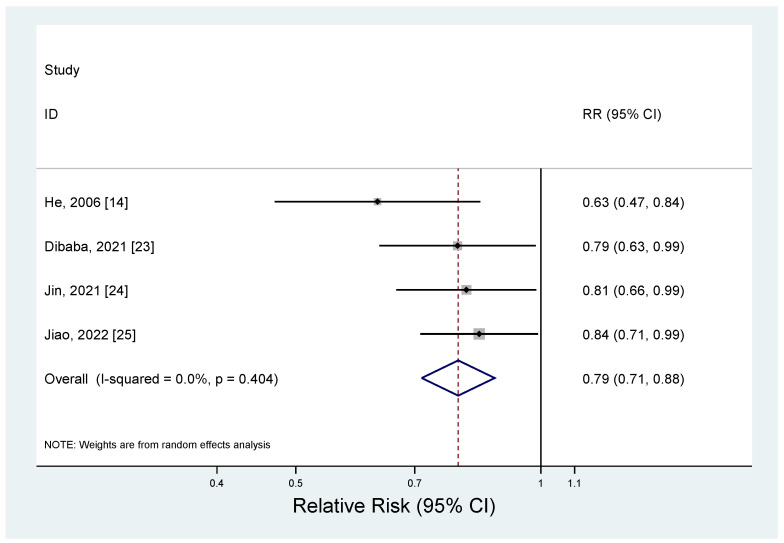
Forest plots of the prospective cohort studies of metabolic syndrome for high versus low magnesium intake [14,23,24,25].

**Figure 3:**
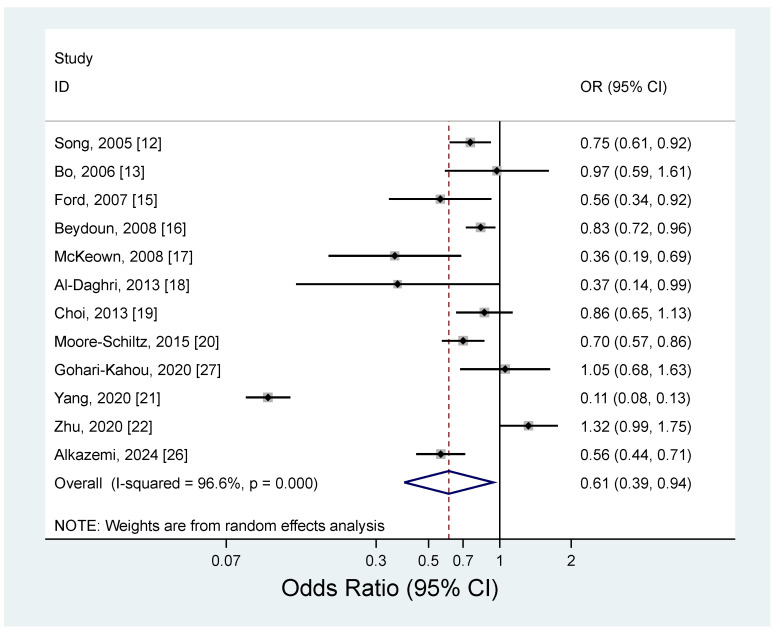
Forest plots of the cross-sectional studies of metabolic syndrome for high versus low magnesium intake [12,13,15,16,17,18,19,20,21,22,26,27].

The results of the meta-analysis regarding the association between magnesium intake and metabolic syndrome by study design are shown in Figure 2 and Figure 3. The pooled RR for the prospective cohort studies was 0.79 (95% CI: 0.71–0.88), with no heterogeneity (*I*^2^ = 0.0%; *p* = 0.40) (Figure 2). For the cross-sectional studies, the pooled OR was 0.61 (95% CI: 0.39–0.94), and a significant heterogeneity was observed among the studies (*I*^2^ = 96.6%; *p* < 0.001) (Figure 3). The observed heterogeneity slightly decreased (*I*^2^ = 71.2%; *p* < 0.001) after excluding a study with the strongest inverse association between dietary intake and metabolic syndrome [21]. The subgroup analysis results for the cross-sectional studies are presented in Table 4. When we examined by sex, a significant inverse association between dietary magnesium and metabolic syndrome was found, but only for women (OR = 0.69; 95% CI: 0.58–0.83); men showed a non-significant inverse association (OR = 0.77; 95% CI: 0.59–1.01). However, the difference by sex was not significant (*p* for difference = 0.55). By geographic region, an inverse association between dietary magnesium and metabolic syndrome was significant in the United States (OR = 0.71; 95% CI: 0.60–0.84), but the difference by region was not significant (*p* for difference > 0.6 for all comparisons). There was no significant difference for the sample size (*p* for difference = 0.73) or adjustment factor (*p* for difference > 0.4 for all comparisons).

**Figure 4:**
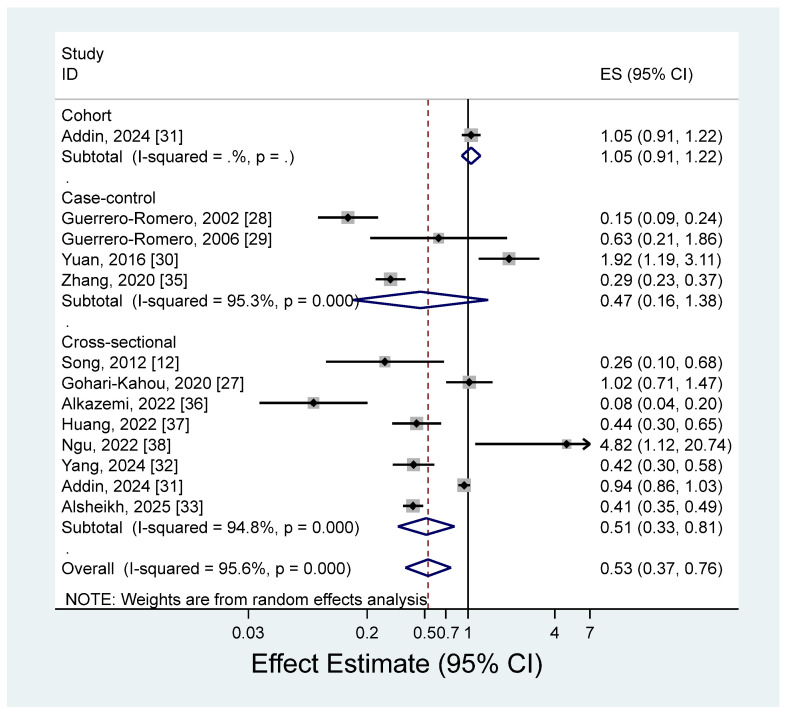
Forest plots of the observational studies of metabolic syndrome for high versus low blood magnesium levels [12,27,28,29,30,31,32,33,35,36,37,38].

The pooled estimate (ES) of metabolic syndrome for the highest versus lowest blood levels of magnesium is presented in Figure 4 (ES = 0.53; 95% CI: 0.37–0.76). There was a significant heterogeneity among the studies (*I*^2^ = 95.6%; *p* < 0.001).

### 3.3. Publication Bias

There was no significant evidence of publication bias in the prospective cohort studies examining the association between dietary magnesium and metabolic syndrome in Begg’s test (*p* = 0.09), but some evidence of publication bias was observed in Egger’s test (*p* = 0.04). We found no significant evidence of publication bias in the cross-sectional studies investigating the association between dietary magnesium and metabolic syndrome (Begg’s test, *p* = 0.24; Egger’s test, *p* = 0.96) or for studies reporting the association between blood levels of magnesium and metabolic syndrome (Begg’s test, *p* = 0.86; Egger’s test, *p* = 0.20).

## 4. Discussion

The current meta-analysis summarized the association between magnesium intake and metabolic syndrome from prospective cohort and cross-sectional studies. A high magnesium intake was associated with a 21% lower incidence of metabolic syndrome compared with a low magnesium intake in the meta-analysis of prospective cohort studies. The results of the meta-analysis of cross-sectional studies showed that people with a high magnesium intake had 39% lower odds of metabolic syndrome compared with people with a low magnesium intake.

The observed inverse association between magnesium intake and metabolic syndrome in our analysis was in line with previous results. A meta-analysis published in 2014, including six cross-sectional studies, suggested that a high magnesium intake was associated with 31% lower odds of metabolic syndrome than a low magnesium intake [39]. In addition, the results of a meta-analysis of two cohort and seven cross-sectional studies indicated that high consumption of magnesium was associated with 27% lower odds of metabolic syndrome [40]. Not only observational studies but also experimental studies have suggested an inverse association between magnesium intake and metabolic syndrome. The summary results of twelve randomized controlled trials that examined the effect of magnesium supplementation on insulin resistance in humans showed a reduction in the homeostasis model assessment of insulin resistance (HOMA-IR) and fasting glucose levels with a 50 mg increment in dietary magnesium [52]. A pooled analysis of eight randomized controlled trials demonstrated that magnesium supplementation contributed to reducing the systolic and diastolic blood pressure of type 2 diabetes patients [53]. A meta-analysis of twelve randomized controlled trials suggested that longer than twelve weeks of magnesium supplementation reduced serum total cholesterol, and magnesium supplementation less than 300 mg decreased serum low-density lipoprotein (LDL) cholesterol levels [54]. In addition, accumulating evidence from twenty-five randomized controlled trials has reported an increase in HDL cholesterol with magnesium supplementation [55]. Regarding obesity, a meta-analysis of thirty-two randomized controlled trials examining obesity measures found a significant reduction in BMI following magnesium supplementation [56]. These protective effects of magnesium intake on components of metabolic syndrome observed through randomized controlled trials may have contributed to the inverse association between magnesium consumption and metabolic syndrome.

Several potential mechanisms can explain the beneficial effect of magnesium on metabolic syndrome. Firstly, magnesium controls the insulin signaling pathway by enhancing tyrosine kinase activity by increasing the insulin receptor’s affinity for adenosine triphosphate [57]. In the downstream pathways, magnesium regulates the membrane transport of the glucose receptor GLUT4 in muscles, acts as an essential regulator of gluconeogenic enzymes (including glucose-6-phosphatase and phosphoenolpyruvate carboxykinase in the liver), and acts as an anti-inflammatory factor that reduces the secretion of interleukin 1 and tumor necrosis factor-α in adipose tissue [58]. As magnesium possesses these functions, hypomagnesemia may lead to increased insulin resistance, which has been confirmed in animal studies [59]. Secondly, magnesium acts as an antagonist of calcium, which acts as a vasoconstrictor molecule while being involved in the secretion of catecholamines from the adrenal glands [60,61]. In the coronary endothelium, magnesium stimulates the production and release of vasodilation molecules, including nitric oxide and prostacyclin [61,62]. Thirdly, magnesium modulates the activity of enzymes such as lipoprotein lipase (LPL), desaturase, and lecithin–cholesterol acyltransferase (LCAT) [63]. LCAT plays an important role in maintaining the lipoprotein balance in the body by lowering LDL cholesterol and triglyceride and raising HDL cholesterol [63,64]. An impairment in LPL and LCAT activity results in increases in triglycerides, LDL cholesterol, and the saturated-to-unsaturated fatty acid ratio as well as decreases in HDL cholesterol [63,65]. Although the mechanisms of an inverse association between magnesium consumption and obesity are unclear, magnesium can reduce the absorption of fat by forming soaps with fatty acids [66]. Magnesium also is related to the secretion of epinephrine, which plays a role in obesity development [67].

Although not included in our meta-analysis, there were observational studies that reported an association between magnesium intake and metabolic syndrome in patients with specific diseases. A prospective cohort study in Iran found a non-significant inverse association between magnesium intake and metabolic syndrome (OR for tertile3 vs. tertile1 = 0.80; 95% CI: 0.16–3.86) among 160 renal-transplant recipients after a 1 year follow-up [68]. A cross-sectional study from Taiwan analyzed the association between magnesium intake and metabolic parameters, and observed a positive association with HDL cholesterol and an inverse association with obesity [69]. However, they failed to find a significant association with metabolic syndrome incidence [69].

We found a significant inverse association between blood levels of magnesium and metabolic syndrome, but there was high heterogeneity among the studies. Although most of the studies reported estimates that were lower than 1, indicating an inverse association between blood magnesium levels and metabolic syndrome, two studies reported a significant positive association, showing estimates that were higher than 1 [30,38]. One study in China showed a positive association between blood magnesium levels and metabolic syndrome (OR for tertile3 vs. tertile1 = 1.92; 95% CI: 1.19–3.11) [30]. This study adjusted for age and sex only, and metabolic syndrome was defined according to Chinese Diabetes Society criteria. Another study from Taiwan showed an estimate greater than 1 (OR for tertile3 vs. tertile1 = 4.82; 95% CI: 1.12–20.73), but had a relatively small sample of 150 subjects [38]. Although the magnesium level in blood is the fastest and most commonly used clinical indicator of magnesium nutritional status, the actual proportion in blood is less than 1% of the total magnesium in the human body [70]. Given the heterogeneity of our study results and the lack of representativeness of serum magnesium levels in assessing magnesium nutritional status, the observed inverse association between serum magnesium levels and metabolic syndrome should be cautiously interpreted. We additionally performed a subgroup analysis based on the median of high blood magnesium levels (0.88 mmol/L) to ascertain if the results varied by level of magnesium intake. However, no significant differences were observed (ES = 0.51, 95% CI: 0.32–0.80 in <median; ES = 0.55, 95% CI: 0.36–0.85 in ≥median).

To the best of our knowledge, this is the first meta-analysis to examine the prospective association between magnesium intake and metabolic syndrome. Prospective design studies have a lower risk of recall bias than retrospective designs, such as case-control and cross-sectional studies, and are relevant to examine for a causal relationship. In addition to a meta-analysis of prospective cohort studies, we also performed a meta-analysis of cross-sectional studies. Additionally, a meta-analysis of blood magnesium levels and metabolic syndrome was conducted to comprehensively understand the effects of magnesium intake and blood levels on metabolic syndrome. Despite these strengths, there are several limitations of this study that should be carefully considered. Firstly, a subgroup analysis of cohort studies for a more in-depth exploration could not be conducted due to the insufficient number of studies. Secondly, measurement errors could have occurred when assessing magnesium intake. However, errors tend to be non-differential; thus, there was little concern that the results were exaggerated. Lastly, most studies in the meta-analysis adjusted for potential confounders, including energy intake, smoking, and alcohol. However, residual confounders should always be considered when understanding the results of observational studies.

## 5. Conclusions

In conclusion, a high magnesium intake was associated with a lower risk of metabolic syndrome in a meta-analysis of prospective cohort studies. This inverse association was also found in the results of cross-sectional studies. A meta-analysis of observational studies analyzing blood mg levels and metabolic syndrome also showed an inverse association. However, the heterogeneity among studies was high, so caution is needed when interpreting the results. The present results from our analysis require further confirmation by future prospective cohort studies and randomized controlled trials.

## Figures and Tables

**Table 1:** Characteristics of prospective cohort studies included in the meta-analysis of magnesium intake and metabolic syndrome.

First Author, Year	Country	Study Name	Follow-Up Years	Age at Baseline (Years)	Sex	Study Size		Adjustment for Covariates
						Subjects	No. of Cases	
He, 2006 [14]	U.S.	The Coronary Artery Risk Development in Young Adults	15	18–30	Male and female	4637	608	Age, sex, race, education, smoking, physical activity, family history of diabetes, alcohol consumption, baseline BMI, and intakes of fiber, polyunsaturated fat, saturated fat, total carbohydrates, and total energy
Dibaba, 2021 [23]	U.S.	The Reasons for Geographic and Racial Differences in Stroke	10	≥45	Male and female	6802	1470	Age, sex, race, region, the interaction between age and race, education, income, smoking, alcohol consumption, physical activity, energy intake, regular aspirin use, calcium intake, and LDL cholesterol
Jin, 2021 [24]	China	The Harbin Cohort Study on Diet, Nutrition and Chronic Non-Communicable Diseases	5.3	20–74	Male and female	6417	1283	Age, sex, drinking, smoking, physical activity, BMI, fat, protein, carbohydrate, fiber, and total energy
Jiao, 2022 [25]	China	China Health and Nutrition Survey	6.1	≥18	Male and female	6104	2024	Age, sex, education, urban and rural areas, income, smoking, drinking, physical activity, energy, dietary fiber, calcium, and BMI

BMI: body mass index; LDL: low-density lipoprotein.

**Table 2:** Characteristics of cross-sectional studies included in the meta-analysis of magnesium intake and metabolic syndrome.

First Author, Year	Country	Study Name	Age at Baseline (Years)	Sex	Study Size		Adjustment for Covariates
					Subjects	No. of Cases	
Song, 2005 [12]	U.S.	Women’s Health Study	≥45	Female	9887	2411	Age, smoking, exercise, total calories, alcohol use, multivitamin use, parental history of myocardial infarction before 60 years, dietary intake of total fat, cholesterol, folate, glycemic load, fiber, and plasma C-reactive protein concentrations
Bo, 2006 [13]	Italy	NA	45–64	Male and female	1653	384	Age, sex, BMI, smoking, alcohol intake, level of physical activity, dietary intake of total calories, total percentage of fat, and dietary intake of fiber
Ford, 2007 [15]	U.S.	The Third National Health and Nutrition Examination Survey (1988–1994)	≥20	Male and female	7669	1981	Age, sex, race or ethnicity, education, smoking, concentration of C-reactive protein, alcohol use, physical activity, family history of early coronary heart disease, use of vitamins or supplements, history of diabetes, percentage of calories as fat, percentage of calories as carbohydrate, fiber intake, and total energy intake
Beydoun, 2008 [16]	U.S.	National Health and Nutrition Examination Survey (1999–2004)	≥18	Male and female	4519	1166	Age, sex, ethnicity, socioeconomic status (education and poverty income ratio), energy intake, and physical activity
McKeown, 2008 [17]	U.S.	NA	≥60	Male and female	535	214	Age, sex, race, educational attainment, marital status, smoking, alcohol intake, exercise, BMI, total energy intake, percentage energy of saturated fatty acid intake, lipid-lowering medication use, and blood pressure medication
Al-Daghri, 2013 [18]	Saudi Arabia	NA	19–60	Male and female	185	72	Age, energy, BMI, and physical activity
Choi, 2013 [19]	South Korea	The Korea National Health and Nutrition Examination Survey	≥19	Male and female	5136	1288	Age, energy intake, and alcohol frequency (only in males)
Moore-Schiltz, 2015 [20]	U.S.	National Health and Nutrition Examination Survey (2001–2010)	≥20	Male and female	9148	3577	Age, sex, ethnicity, education, household income, total energy intake, fiber intake, and calcium intake
Gohari-Kahou, 2020 [27]	Iran	Mashhad Stroke and Heart Atherosclerotic Disorder study	35–65	Male and female	853	269	Age, sex, and BMI
Yang, 2020 [21]	China	The China Health and Nutrition Survey	≥18	Male and female	8120	2168	Age, sex, total energy intake, smoking, current alcohol consumption, education level, residence, and intake of protein, carbohydrate, and fat
Zhu, 2020 [22]	China	China Nutrition and Health Survey and Shanghai Diet and Health Survey	≥18	Male and female	5323	1836	Age, sex, region, years of education, physical activity level, intended physical exercises, smoking, alcohol use, daily energy intake, iron, and zinc
Alkazemi, 2024 [26]	Kuwait	NA	18–65	Female	170	41	Age, regular menses, and BMI

BMI: body mass index; NA: not applicable.

**Table 3:** Characteristics of observational studies included in the meta-analysis of blood levels of magnesium and metabolic syndrome.

First Author, Year	Country	Study Design	Study Name	Age at Baseline (Years)	Sex	Study Size		Adjustment for Covariates
						Subjects	No. of Cases	
Addin, 2024 [31]	Germany	Cohort	KORA (Cooperative Health Research in the Region of Augsburg) Survey	32–81	Male and female	1358	232	Age, sex, smoking, physical activity, alcohol consumption, serum potassium, and diuretic medication
Guerrero-Romero, 2002 [28]	Mexico	Case-control	NA	42.3 (cases) 41.5 (controls)	Male and female	384	192	Age and the homeostasis model assessment of insulin resistance (HOMA-IR) index
Guerrero-Romero, 2006 [29]	Mexico	Case-control	NA	44.0 (cases) 43.0 (control)	Male and female	441	147	Age, sex, BMI, waist-to-hip ratio, total adiposity, C-reactive protein, and malondialdehyde
Yuan, 2016 [30]	China	Case-control	NA	64.0 (cases) 64.1 (controls)	Male and female	408	204	Age and sex
Zhang, 2020 [35]	China	Case-control	NA	60.1 (cases) 59.9 (controls)	Male and female	4134	2095	Age, sex, education level, smoking, alcohol intake, BMI, physical activity, and family history of disease
Song, 2012 [34]	South Korea	Cross-sectional	NA	37.6; mean	Male and female	514	35	Age, sex, exercise, smoking, and drinking
Gohari-Kahou, 2020 [27]	Iran	Cross-sectional	Mashhad Stroke and Heart Atherosclerotic Disorder Study	35–65	Male and female	853	269	Age, sex, and BMI
Alkazemi, 2022 [36]	Kuwait	Cross-sectional	Al-Addan Hospital and Mubarak Al-Kabeer Hospital	18–65	Male and female	231	51	Age, nationality, education level, employment status, total annual income, menstrual cycle (for women), BMI, and physical activity
Huang, 2022 [37]	China	Cross-sectional	The Eighth Affiliated Hospital of Sun Yat-Sen University	59.22; mean	Male and female	1274	149	Age, sex, smoking, drinking, and estimate glomerular filtration rate
Ngu, 2022 [38]	Taiwan	Cross-sectional	NA	20–64	Male and female	150	40	Age, gender, BMI, and smoking
Yang, 2024 [32]	China	Cross-sectional	China Nutrition and Health Surveillance 2015–2017	≥45	Male and female	2101	NA	Age, sex, education, nationality, area, residence, BMI, and heart rate
Addin, 2024 [31]	Germany	Cross-sectional	KORA (Cooperative Health Research in the Region of Augsburg) Survey	32–81	Male and female	2609	817	Age, sex, smoking status, physical activity, alcohol consumption, serum potassium, and diuretic medication
Alsheikh, 2025 [33]	Qatar	Cross-sectional	Qatar Biobank Study	≥20	Male and female	9389	1929	Age, sex, smoking, physical activity, diet, and education

BMI: body mass index; NA: not applicable.

**Table 4:** Summary of adjusted odds ratios (ORs) of metabolic syndrome for magnesium intake.

Factor	No. of Studies	OR	95% CIs	*p* for Difference
All studies	12	0.61	0.39–0.94	
Stratified by sex				
Male	2	0.77	0.59–1.01	0.55
Female	4	0.69	0.58–0.83	
Stratified by geographical region				
U.S.	5	0.71	0.60–0.84	
Asia	3	0.49	0.10–2.53	0.67 ^a^
Middle East	3	0.66	0.39–1.11	0.99 ^a^
Europe	1	0.97	0.59–1.61	0.63 ^a^
Sample size				
≥Median	6	0.57	0.28–1.15	0.73
<Median	6	0.69	0.51–0.95	
Adjustment for smoking				
Yes	6	0.52	0.20–1.33	0.46
No	6	0.75	0.62–0.89	
Adjustment for alcohol				
Yes	7	0.56	0.25–1.24	0.65
No	5	0.72	0.58–0.89	

OR: odds ratio. ^a^ *p*-value for difference in ORs for Asia vs. U.S. (*p* = 0.67), Middle East vs. U.S. (*p* = 0.99), and Europe vs. U.S. (*p* = 0.63).

## Data Availability

Data are available from the corresponding author upon reasonable request.

## Abbreviations

The following abbreviations are used in this manuscript:

DNA	Deoxyribonucleic acid
HDL	High-density lipoprotein
CVD	Cardiovascular disease
RR	Relative risk
OR	Odds ratio
ES	Estimate
CI	Confidence interval
BMI	Body mass index
LDL	Low-density lipoprotein
LPL	Lipoprotein lipase
LCAT	Lecithin–cholesterol acyltransferase
